# Lysophospholipid remodeling mediated by the LplT and Aas protein complex in the bacterial envelope

**DOI:** 10.1016/j.jbc.2024.107704

**Published:** 2024-08-20

**Authors:** Wei Niu, Trung Vu, Guangwei Du, Mikhail Bogdanov, Lei Zheng

**Affiliations:** 1 Department of Biochemistry and Molecular Biology, Center for Membrane Biology, University of Texas Health Science Center at Houston McGovern Medical School, Houston, Texas, USA; 2 Department of Integrative Biology and Pharmacology, University of Texas Health Science Center at Houston McGovern Medical School, Houston, Texas, USA

**Keywords:** transporter, lysophospholipid, acyltransferase, protein interaction, membrane

## Abstract

Lysophospholipid transporter LplT and acyltransferase Aas consist of a lysophospholipid-remodeling system ubiquitously found in gram-negative microorganisms. LplT flips lysophospholipid across the inner membrane which is subsequently acylated by Aas on the cytoplasmic membrane surface. Our previous study showed that the proper functioning of this system is important to protecting *Escherichia coli* from phospholipase-mediated host attack by maintaining the integrity of the bacterial cell envelope. However, the working mechanism of this system is still unclear. Herein, we report that LplT and Aas form a membrane protein complex in *E. coli* which allows these two enzymes to cooperate efficiently to move lysophospholipids across the bacterial membrane and catalyze their acylation. The direct interaction of LplT and Aas was demonstrated both *in vivo* and *in vitro* with a binding affinity of 2.3 μM. We found that a cytoplasmic loop of LplT adjacent to the exit of the substrate translocation pathway plays an important role in maintaining its interaction with Aas. Aas contains an acyl-acyl carrier protein synthase domain and an acyl-transferase domain. Its interaction with LplT is mediated exclusively by its transferase domain. Mutations within the three loops near the putative catalytic site of the transferase domain, respectively, disrupt its interaction with LplT and lysophospholipid acylation activity. These results support a hypothesis of the functional coupling mechanism, in which LplT directly interacts with the transferase domain of Aas for specific substrate membrane migration, providing synchronization of substrate translocation and biosynthetic events.

Phospholipids are the major component of cell membranes in the bacterial envelope. Bacterial membranes primarily consist of diacyl-form lipids. In *Escherichia coli*, membrane lipids are composed of 70% phosphatidylethanolamine (PE), 20% phosphatidylglycerol (PG), and 5 to 10% cardiolipin (1). This composition is kept at relatively constant levels since maintaining phospholipid homeostasis is important to stabilize the membrane bilayer structure and achieve proper membrane activities (2).

In contrast to those membrane bulk lipids, lysophospholipids only have one acyl chain at either the *sn*-1 or *sn*-2 position of the glycerol backbone. They are usually present in bacterial cell envelopes in very small amounts (<1%) but can be accumulated substantially under certain environmental conditions (3, 4). For example, the pathogenic bacterium *Vibrio cholerae* remodels its lipid profile and produces an anomalously high amount of lyso-phosphatidylethanolamine (lyso-PE) accounting for ∼30% of the total lipid composition after exposure to bile salts which may occur in the early stages of infection or during growth in the presence of ocean sediment (5, 6). In gram-negative bacteria, lysophospholipids are mainly generated as metabolic intermediates in phospholipid synthesis or from membrane degradation occurring in both the inner membrane and outer membrane (Fig. 1). In the inner membrane, the process of cell wall biosynthesis constitutively produces lysophospholipids during the lipoprotein maturation process, *i.e.*, lipoprotein acyltransferase (Lnt) transfers the *sn*-1 acyl chain from a PE to the N terminus of the major outer membrane precursor (Lpp), generating a triacylated mature Lpp and lyso-PE as by-product (7). Lyso-PE can also be generated by various pathways as stress responses in the outer membrane. Upon outer membrane damage or stress, activated PldA hydrolyzes PE (8) whereas lipid A palmitoyltransferase PagP catalyzes the transfer of a palmitoyl group from a PE to lipid A to generate hepta-acylated lipid A (9). Both reactions would inevitably release lyso-PE into the bacterial envelope.

**Figure 1:**
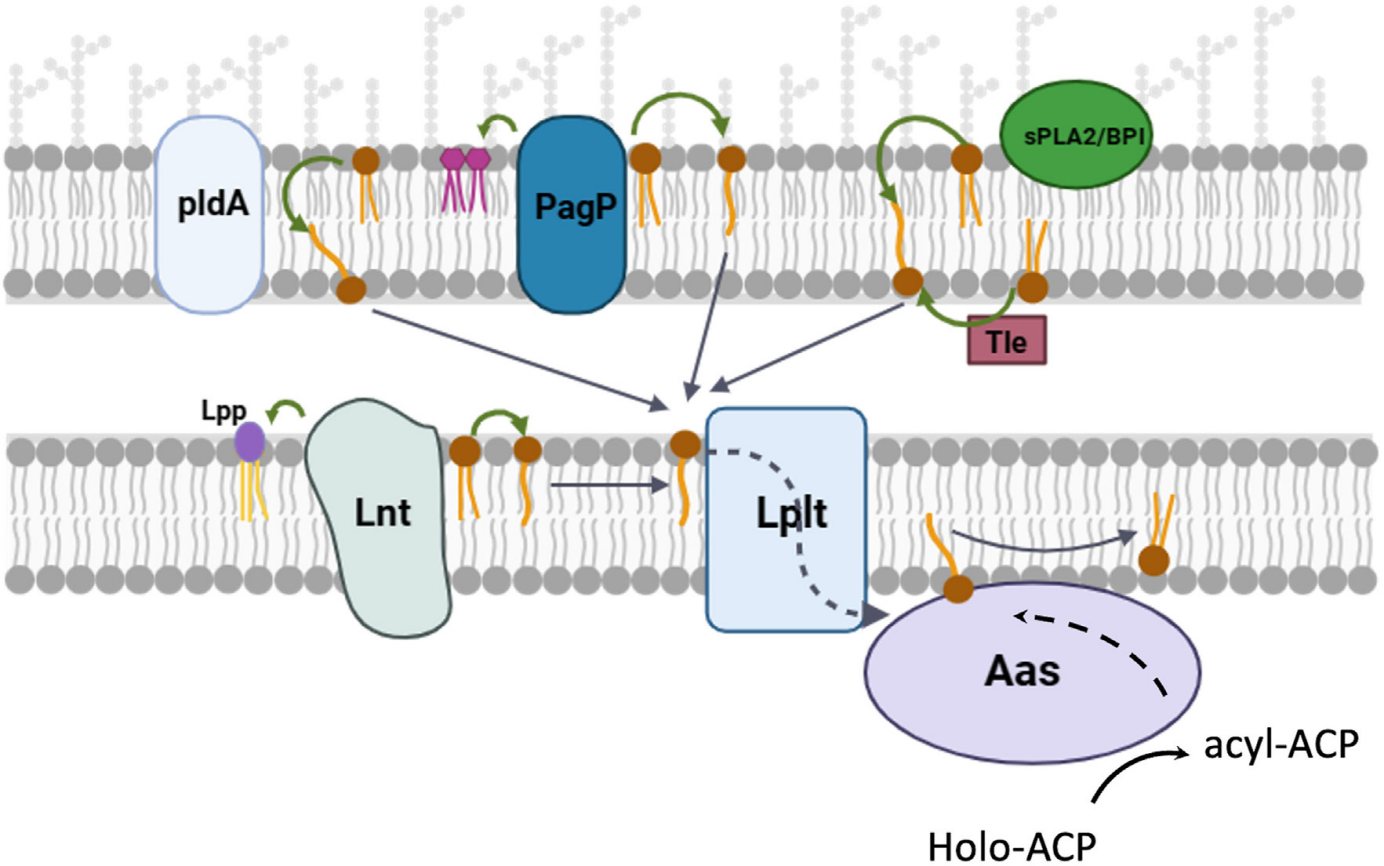
**The functional coupling of LplT and Aas proteins.** Lysophospholipids are generated by multiple endogenous and exogenous lipid degradation reactions in both the outer and inner membranes of *Escherichia coli*. Several phospholipases including endogenous PldA and exogenous PLA_2_ from the host and secreted phospholipase Tle from other bacteria hydrolyze membrane phospholipids to generate lysophospholipids in the outer membrane; lipid A palmitoyltransferase PagP and lipoprotein acyltransferase Lnt catalyze acyl transfer from a PE to lipid A or lipoprotein, releasing lyso-PE as by-product to the bilayer. LplT flips lysophospholipids from the periplasmic space or the outer leaflet of the inner membrane. Aas is a peripheral membrane enzyme performing a two-step reaction. In the first step, the synthetic domain of Aas synthesizes acyl-acyl carrier protein (acyl-ACP) using holo-ACP as substrate; in the second step, newly synthesized acyl-ACP is used by the acyl transferase domain as an acyl donor to convert lysophospholipids to diacyl counterparts. ACP, acyl carrier protein; PE, phosphatidylethanolamine; PLA_2_, phospholipase A.

Lysophospholipids may also be generated from membrane degradation mediated by exogenous phospholipases as a potent antibacterial strategy. In polymorphonuclear neutrophils, secreted phospholipase A_2_ (PLA_2_) is delivered into the ingested bacterial cells assisted by bacterial permeability protein, causing phospholipid degradation and consequent membrane disorganization and cell disassembly (10, 11). In addition, lysophospholipids may be generated during interbacterial antagonistic interactions. Microorganisms can deliver a new class of phospholipase A protein (Tle) to different bacterial species *via* the type VI secretion system to decompose PE (12). Although lysolipids are minor lipids in *Escherichia coli*, they may induce profound effects largely due to their detergent-like properties. Accumulation of lysophospholipids markedly disrupts membrane structure evidenced by increasing membrane permeability and inducing membrane curvature (13, 14). To minimize these disruptive effects, lysophospholipids must be eliminated or modified to restore impaired membranes to their normal bilayer structure.

LplT and Aas contribute to the major mechanisms for remodeling lysophospholipids in *E. coli* and possibly other gram-negative bacteria. LplT is a lysophospholipid transporter protein located in the inner membrane (15). LplT promotes an energy-independent flipping of multiple species of lysolipids including lyso-PE, lysophosphatidylglycerol (lyso-PG), and diacyl or deacylated cardiolipin derived from both the periplasmic space and the outer leaflet of the inner membrane with comparable efficiency (16). Aas is a bifunctional lysophospholipid acyltransferase/acyl-acyl carrier protein synthetase peripherally located on the cytoplasmic surface (17, 18). Aas synthesizes acyl-acyl carrier protein (acyl-ACP) using holo-ACP and then uses acyl-ACP as an acyl donor to achieve the acylation of lysophospholipids to generate diacyl PE, diacyl PG, or triacyl cardiolipin on the inner leaflet of the membrane (Fig. 1). We have found that the inactivation of the LplT or Aas abolished bacterial lysophospholipid acylation activity and reduced membrane packing and disrupted lipid asymmetry with more PE lipids present in the outer leaflet of the outer membrane (19). These changes drastically increased bacterial susceptibility to the combined actions of inflammatory fluid components and secreted PLA_2_, suggesting an important role of LplT and Aas in the mechanisms of bacterial defense against host innate immune attack.

It is still unclear how these two proteins are coordinated with each other to achieve effective lysophospholipid uptake and acylation. The coupling of these proteins is so far only supported by genetic evidence that LplT and Aas are adjacently encoded by the same bicistronic operon in many gram-negative bacteria, including *E. coli* and *Klebsiella pneumoniae* (15). However, in some other bacteria, the genes encoding LplT and Aas are fused to a single ORF apparently. However, the functions of these proteins remain uncharacterized.

In this study using both *in vivo* and *in vitro* biochemical approaches, we were able to establish an efficient coupling mechanism of this lysophospholipid remodeling system by demonstrating that LplT and Aas form a functional membrane protein complex. We also identified key structural components of LplT and Aas involved in the protein complex formation. Our data suggest that the interaction of LplT and Aas establishes a substrate-tunneling mechanism, in which the catalytic sites of two proteins are interconnected to facilitate the substrate specificity and efficiency of lysolipid acylation. This study establishes a model that explains how the LplT-Aas system contributes to the maintenance of the integrity of the membrane bilayer in the bacterial envelope.

## Results

**Figure 2:**
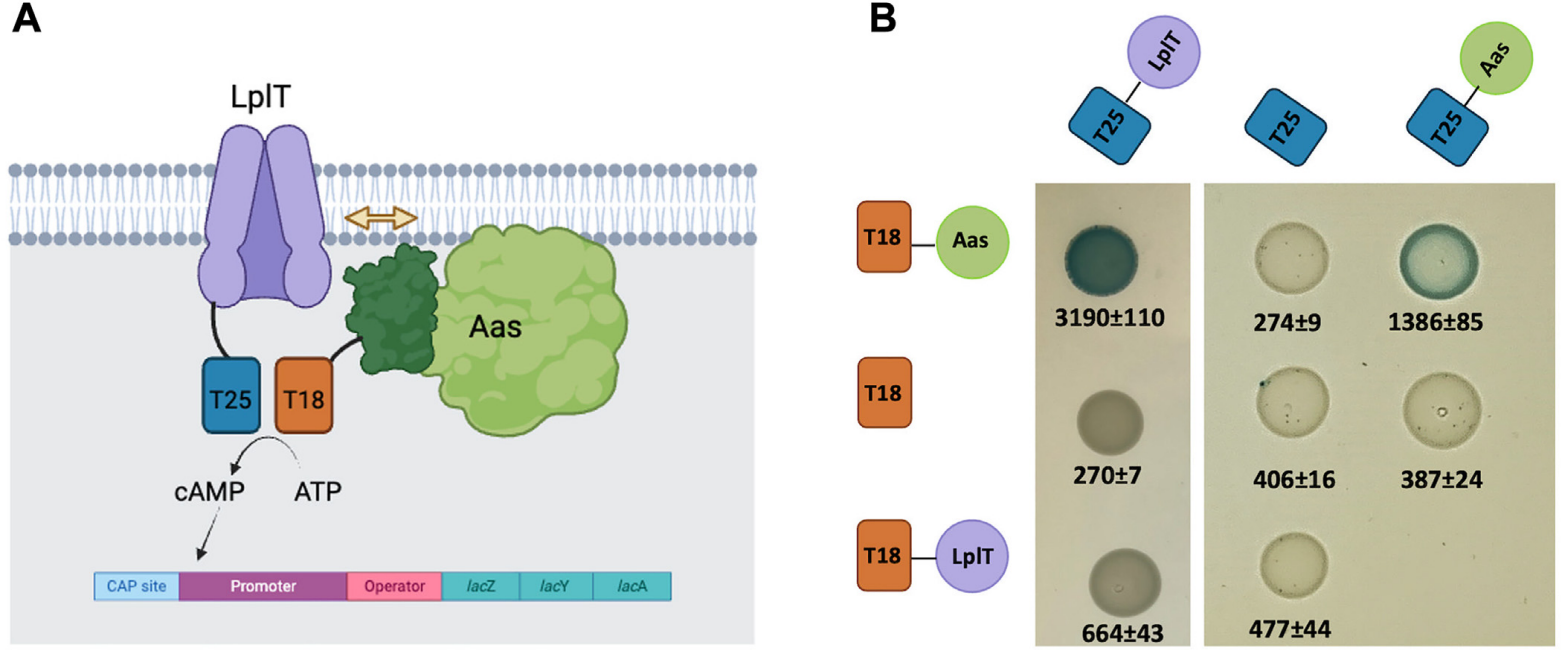
***In vivo* characterization of LplT-Aas interaction.***A*, cartoon illustration of the BACTH approach to investigate the LplT-Aas interaction. The interaction of LplT and Aas induces the association of the N terminally tagged T25 and T18 domains of *Bordetella pertussis* adenylate cyclase, which is detected by β-galactosidase activity. *B*, *Escherichia coli* BTH101 cells expressing the indicated fusion proteins were spotted onto Lucia broth X-gel agar and grown for 48 h at 30 °C. *Blue spots* indicate protein-protein interaction, whereas *white spots* indicate weak to no protein interaction. Images from two plates are separated by a spacer. For each cotransformant, the interaction was quantified by β-galactosidase assays as nmoles of β-galactose formed per minute per mg of the lysate. The values represent the mean and standard deviation (n = 3). BACTH, Bacterial Adenylate Cyclase-based Two-Hybrid.

Protein-protein interactions are crucial for achieving biological activities. We hypothesized that LplT and Aas assemble a membrane protein complex. Structural and functional complementarities in both protein-protein interactions and protein-ligand binding is one of the most fundamental determinants of molecular interactions. However, many membrane protein interactions occur transiently in the lipid bilayer, it may be challenging to maintain such spatiotemporal interactions *in vitro* in detergent-solubilized states. To test the hypothesis, we first adopted the Bacterial Adenylate Cyclase-based Two-Hybrid (BACTH) approach to explore any potential interaction between LplT and Aas in their native membrane environments. BATCH is a genetic approach that probes any protein-protein interactions in *E. coli* based on the *in vivo* reconstitution of the catalytically active domain of *Bordetella pertussis* adenylate cyclase (CyaA) from T18 and T25 fragments (20). In the assay, LplT and Aas were fused with the N-terminus of the T18 or T25 domain, respectively, for coexpression in the *E. coli* BTH101 host strain (Fig. 2*A*). If LplT and Aas interact, the fused T18 and T25 domains will be brought close (dimerized), resulting in cyclic AMP (cAMP) production. Therefore, the protein interaction of LplT and Aas can be assessed by the cAMP-regulated expression of reporter gene LacZ. As seen in Figure 2*B*, the coexpression of T25-LplT and T18-Aas yielded a dark-blue colony due to highly stimulated β-galactosidase activity. This suggested that the coexpression of two fusions mediates the reconstitution of the CyaA catalytic domain caused by the specific interaction of LplT and Aas. Supporting this conclusion tagged T25-LplT or T18-Aas coexpressed with only T18 or T25 domains, respectively, did not stimulate β-galactosidase activity. These assays also suggested that Aas itself forms a dimer or oligomer since the coexpression of T25-Aas and T18-Aas generated blue colonies, whereas only white colonies were observed when T25-LplT and T18-LplT were coexpressed. These results support our hypothesis that LplT and Aas form a membrane protein complex in the *E. coli* inner membrane.

**Figure 3:**
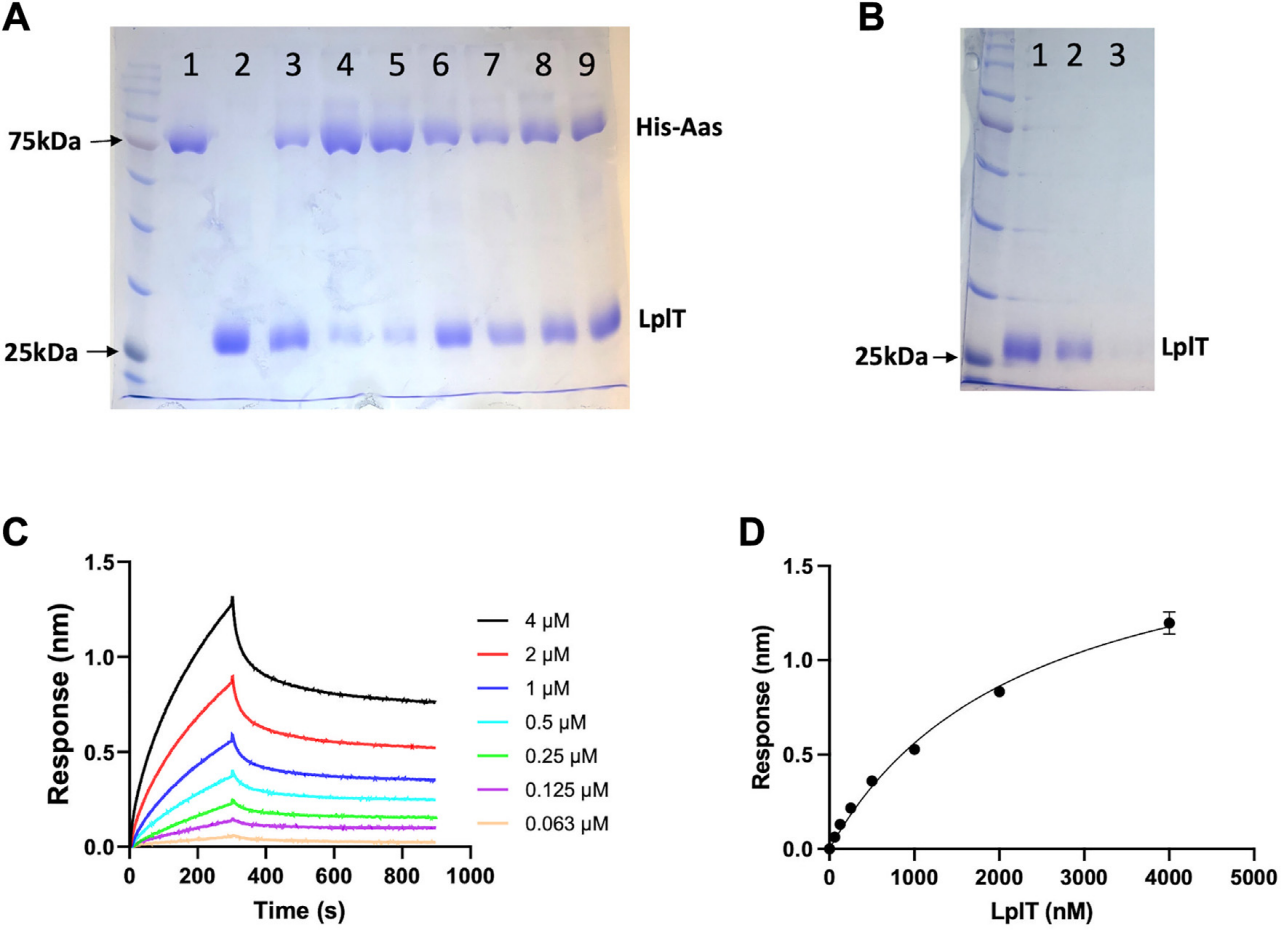
***In vitro* characterizations of LplT-Aas interaction.***A*, SDS-PAGE analysis of the pull-down assays using purified LplT and Aas proteins on Ni-NTA beads. Lane 1: 6× His tagged Aas (bait); lane 2, tag-free LplT (prey); lanes 3 to 9 are eluted from the reactions in the presence of detergent DDM only (lane 3), lyso-PE (lane 4), lyso-PG (lane 5), POPE (lane 6), POPG (lane 7), cardiolipin (lane 8), and *Escherichia coli* total polar lipid extract (lane 9), respectively. *B*, pull-down assay negative control. Tag-free LplT (lane 1) passed through Ni-NTA beads (lane 2) and showed no binding in the imidazole elute (lane 3). The gels were stained with Coomassie blue. *C*, representative profiles of biolayer interferometry (BLI) measuring His-Aas binding to LplT tag-free proteins. His-Aas was loaded onto the Ni-NTA biosensors. LplT flowed in the reactions from 62.5 to 4000 nM as indicated. After 300s, dissociation was triggered by moving the sensors in a buffer. *D*, kinetic analysis of the LplT binding to Aas shown in C for equilibrium *K*_D_ determination. Data were fitted into the nonlinear regression curve using GraphPad 10. Error bars represent standard deviations (n = 3). DDM, n-dodecyl β-D-maltoside; lyso-PG, lysophosphatidylglycerol; PE, phosphatidylethanolamine; POPE, 1-palmitoyl-2-oleoyl-sn-glycero-3-phosphoethanolamine; POPG, 1-palmitoyl-2-oleoyl-sn-glycero-3-phosphoglycerol.

Although the BACTH assays suggest the existence of the interaction of LplT and Aas, it is also possible that their interaction is mediated by other endogenous components in the *E. coli* membrane. To exclude this possibility, the interaction of LplT and Aas was tested directly using purified proteins. To perform pull-down assays, the LplT and N terminally 6His-tagged Aas proteins from *E. coli* were expressed, solubilized using n-dodecyl β-D-maltoside (DDM), and purified to homogeneity (Fig. 3*A*). The Aas protein was then immobilized on Ni-NTA beads to allow pull-down assays using tag-free LplT protein. The results clearly showed that the LplT protein only binds to the beads in the presence of Aas, not in its absence (Fig. 3, *A* and *B*), confirming the direct protein interaction between LplT and Aas. We also asked whether the protein interaction is affected by any lipid molecules since both LplT and Aas proteins are membrane-residing lipid enzymes. Interestingly, our results indicate that the LplT-Aas interaction was noticeably reduced in the presence of their substrates, lyso-PE, and lyso-PG, based on the relative intensity of LplT/Aas on SDS-PAGE (Fig. 3*B*). This reduction was not observed with their diacyl counterparts at the same concentrations, *i.e.*, the presence of 2 mM 1-palmitoyl-2-oleoyl-sn-glycero-3-phosphoethanolamine (POPE), 1-palmitoyl-2-oleoyl-sn-glycero-3-phosphoglycerol (POPG), cardiolipin, and *E. coli* total polar lipid extract resulted in no changes in LplT binding. This effect is unlikely attributed to any detergent effect of lysophospholipids since lipids were prepared in a solution containing a high concentration (20× excess) of DDM detergent. These results suggest a specific role of lysophospholipid ligands in regulating the LplT-Aas interaction.

The binding kinetics was characterized using biolayer interferometry analysis (BLI). Like the pull-down assays, BLI was performed on Ni-NTA biosensors immobilized with purified Aas protein. BLI provides a real-time measurement of protein binding on the sensor surface by detecting the interference of reflected white light. As seen in Figure 3*C*, adding the LplT protein onto the Aas-sensors induced increases in light interference signals as indicated by wavelength shifts, indicative of a binding event occurring between the two proteins. After equilibration for 300 s, the dissociation was triggered by moving the sensors to a buffer as observed by the signal decrease. BLI data measured at varied protein concentrations (62.5–4000 nM) were fitted well to the nonlinear decay kinetics model in a stoichiometry of 1:1, yielding a binding affinity (*K*_D_) of 2.3 μM (Fig. 3*D*). These results further confirm that LplT and Aas form a stable protein complex in the membrane bilayer.

We then asked how LplT and Aas form a complex since these two proteins have distinct membrane topologies and residencies, *i.e.*, LplT is an integral membrane protein and contains no soluble domains whereas Aas is a peripheral membrane protein and does not have any predicted transmembrane helices. One possibility is that the N terminus of LplT interacts with the C terminus of Aas as suggested by those tandem-fused LplT-Aas homologs found in some bacteria. However, this possibility can be ruled out since (1) the fusion of Aas at the C terminus of LplT disrupted LplT expression, and (2) the truncation of 15 amino acid residues at the N-terminus of Aas resulted in no change in protein interaction (as will demonstrated below). Therefore, we hypothesized that the interaction of LplT and Aas is mediated by their specific protein conformations. The above assays prove that BACTH is an useful approach in examining the LplT-Aas protein interaction in the native membrane environment. Therefore, we used this assay to further characterize the complex formation.

**Figure 4:**
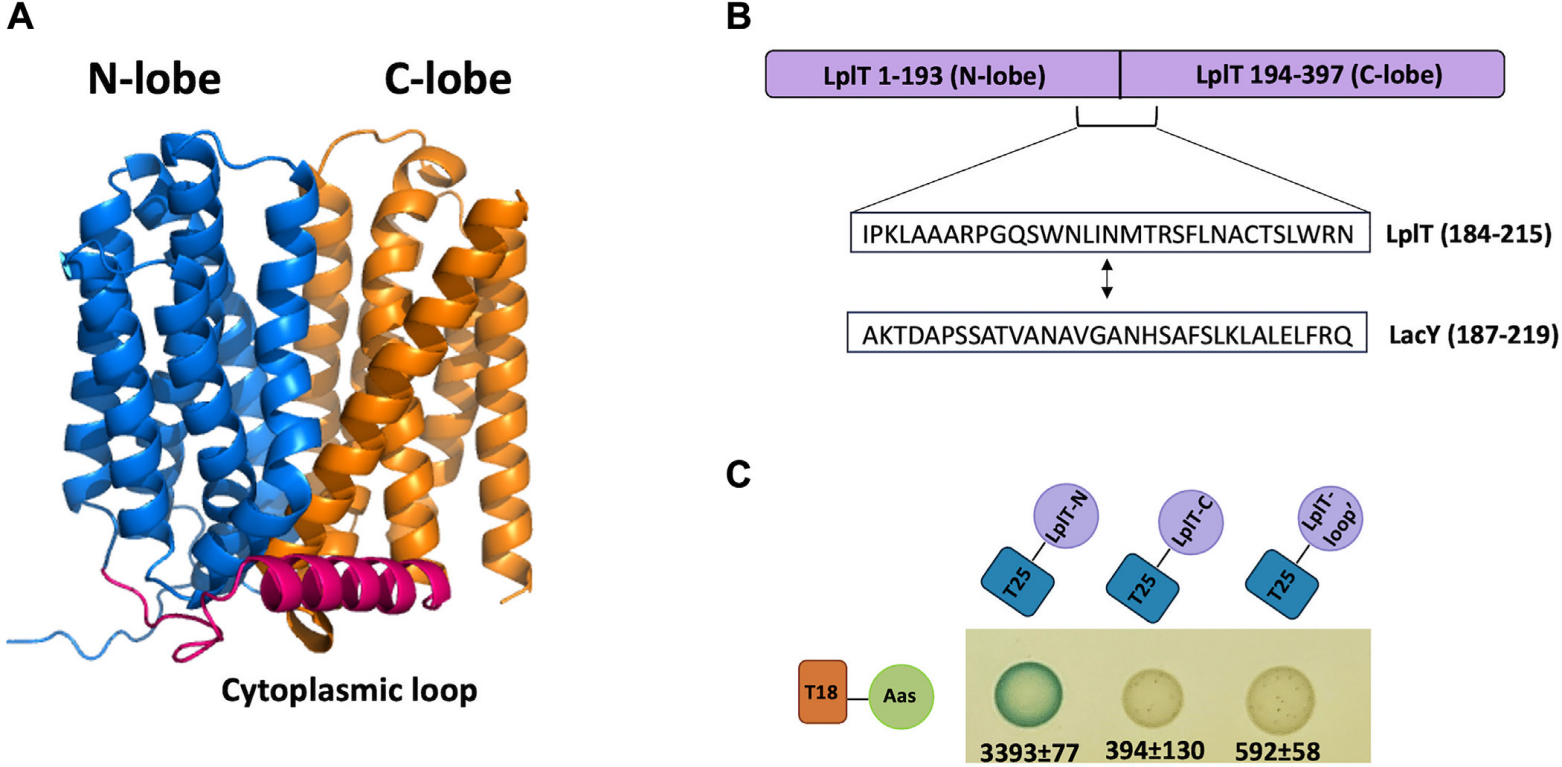
**Key structural components of LplT in protein interaction.***A*, Alphafold-predicted structural model of LplT from *Escherichia coli* consists of an N-lobe (*blue*) and a C-lobe (*orange*), which are linked by a large cytoplasmic loop (*red*). *B*, the swapped sequence of the central cytoplasmic loop between LplT and LacY. *C*, BACTH assays. *E. coli* BTH101 cells expressing T18-Aas and T25-fused LplT mutants as indicated were spotted onto Lucia broth X-gel agar and grown for 48 h at 30 °C. *Blue spots* indicate protein-protein interaction, whereas *white spots* indicate weak to no protein interaction. For each cotransformant, the interaction was quantified using β-galactosidase assays as nmoles of β-galactose formed per minute per mg of the lysate. The values represent the mean and standard deviation (n = 3). BACTH, Bacterial Adenylate Cyclase-based Two-Hybrid.

LplT is a member of the major facilitator superfamily (MFS), which shares a conserved structural fold of twelve transmembrane helices with a pseudo-two-fold symmetry which can be seen as an interlocking inverted-topology repeat of two lobes of six transmembrane a-helices each lining a central transport pathway (Fig. 4, *A* and *B*). In our previous LplT modeling study, two helical bundles, Helix 1 to 6 and 7 to 12, form N-lobe (amino acid residues 1–193) or C-lobe (amino acid residues 194–397) to assemble a substrate translocation pathway on the domain interface (21). We found that the N-lobe is sufficient to maintain the interaction with Aas whereas its truncation abolished the protein interaction (Fig. 4*C*). It is worth noting that LplT lacks any known signal sequences. Therefore, the truncations are unlikely to alter the membrane localization of LplT. Aas is located on the cytoplasmic surface of the inner membrane. Its interaction with LplT is anticipated to occur on the inner membrane leaflet. In LplT, the N-lobe and C-lobe are connected by a large cytoplasmic loop of ∼30 amino acid residues (K184-N215) between TM6 and TM7 (Fig. 4*B*). We found that the substitution of this loop with the counterpart of LacY (residues 187–219), another MFS member from *E. coli*, completely abolished the LplT interaction with Aas. These results reveal that the N-lobe and cytoplasmic loop are important for the interaction of LplT with Aas.

**Figure 5:**
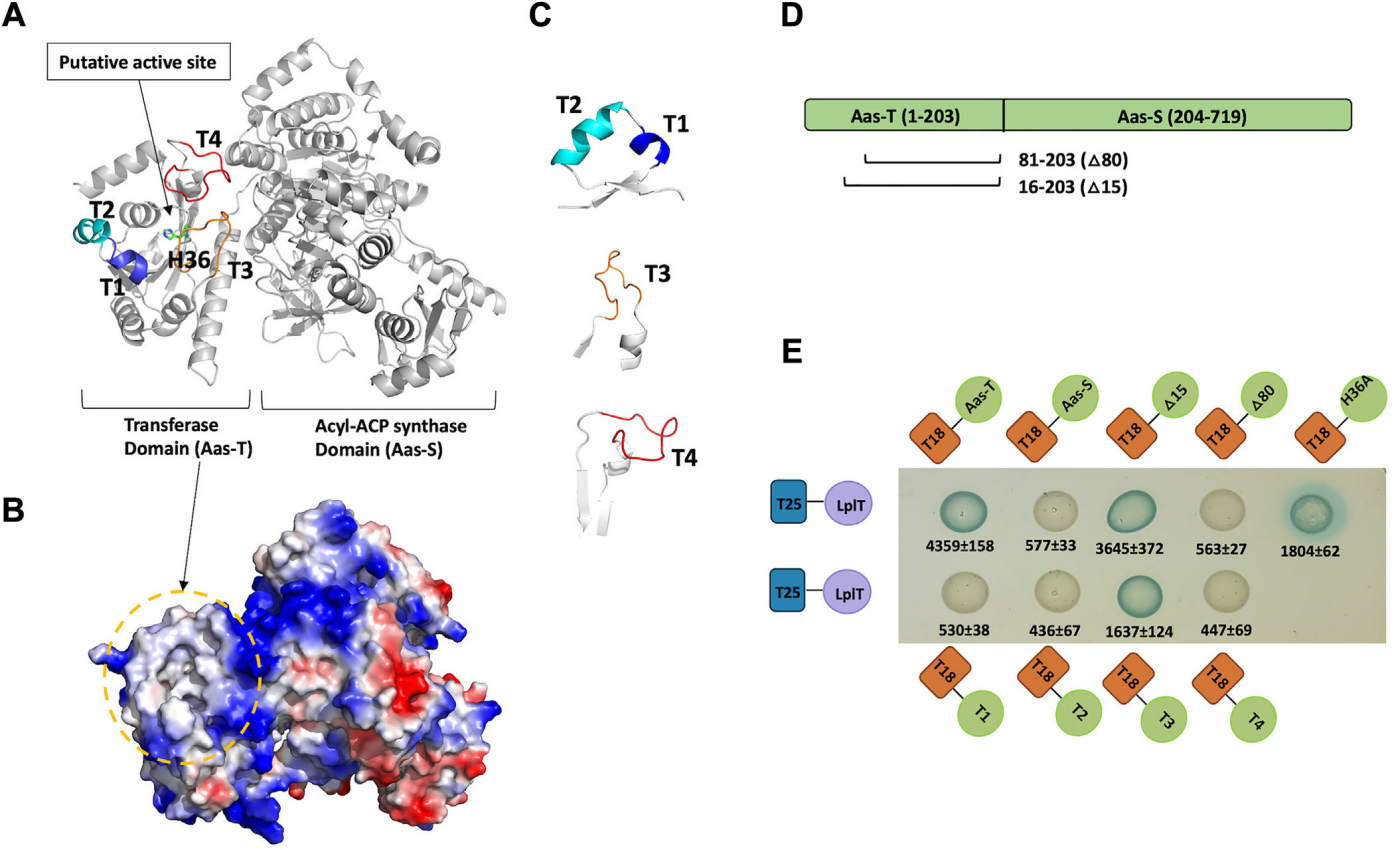
**Structural determinants of Aas interaction.***A*, Alphafold-predicted model shows two catalytic domains of Aas from *Escherichia coli*. The putative active site of the acyl transferase domain is indicated using an arrow. The four loops, T1 (*blue*), T2 (*cyan*), T3 (*orange*), and T4 (*red*), are located at the entry of the active site. *B*, electrostatics map of the Aas structural model generated by PyMOL showing positively charged residues (*blue*), negatively charged residues (*red*), and hydrophobic residues (*gray*). *C*, the local conformation of T1 (*blue*), T2 (*cyan*), T3 (*orange*), and T4 (*red*). *D*, the location of the Aas mutations. *E*, BACTH assays. *E. coli* BTH101 cells expressing T25-LplT and T18-Aas variants as indicated were spotted onto Lucia broth X-gel agar and grown for 48 h at 30 °C. *Blue spots* indicate protein interaction, whereas *white spots* indicate weak to no protein interaction. For each cotransformant, the interaction was quantified in β-galactosidase assays as nmoles of β-galactose formed per minute per mg of the lysate. The values represent the mean and standard deviation (n = 3). BACTH, Bacterial Adenylate Cyclase-based Two-Hybrid.

Aas is a bifunctional enzyme consisting of an N-terminal transferase domain (designated as Aas-T, amino acid residues 1–203), and a C-terminal acyl-ACP synthase domain (designated as Aas-S, amino acid residues 204–719) (Fig. 5*A*). The BACTH assays suggest that the Aas-T domain is critical to maintaining its interaction with LplT since the truncation of the Aas-T domain abolished its interaction with LplT whereas the coexpression of T25-LplT and T18-Aas-T yielded dark blue colonies (Fig. 5*E*). Although the truncation of the N-terminal 15 amino acid residues (M1-R15) of Aas-T resulted in no change in the LplT-Aas interaction, we found that truncating a larger fragment (M1-D80), interrupted its interaction with LplT (Fig. 5, *D* and *E*). Within this fragment, H36 is the key residue for the acyl transferase reaction. Rock group has previously shown that the mutation of H36A abolished the lysolipid acylation activity of Aas (22). However, it is unlikely that the acylation activity of Aas plays any significant role in the complex formation interaction since the mutation of H36A did not alter the protein-protein interaction. Thus, we hypothesized then that the Δ80 amino acid truncation disrupts the structural conformation of the catalytic site since H36 is located in a putative substrate binding pocket based on the Alphafold model of Aas (https://www.uniprot.org/uniprotkb/P31119/entry#structure) (23) (Fig. 5*A*). The pocket is formed by two small tandem helices, T1 (T59-Q64) and T2 (W65-I74), and two short loops, T3 (P104-K116) and T4 (T143-R155) (Fig. 5*C*). The truncation of T1, T2, or T4, respectively, diminished the interaction with LplT in contrast to no changes observed with the T3 truncation (Fig. 5*D*). It is worth noting that the truncations were designed carefully based on the Alphafold model to prevent any significant alteration of the structural conformation (Fig. 5*C*). These results suggest that the catalytic pocket of the transferase domain, perhaps T1, T2, and T4 fragments, is involved in the Aas interaction with LplT.

**Figure 6:**
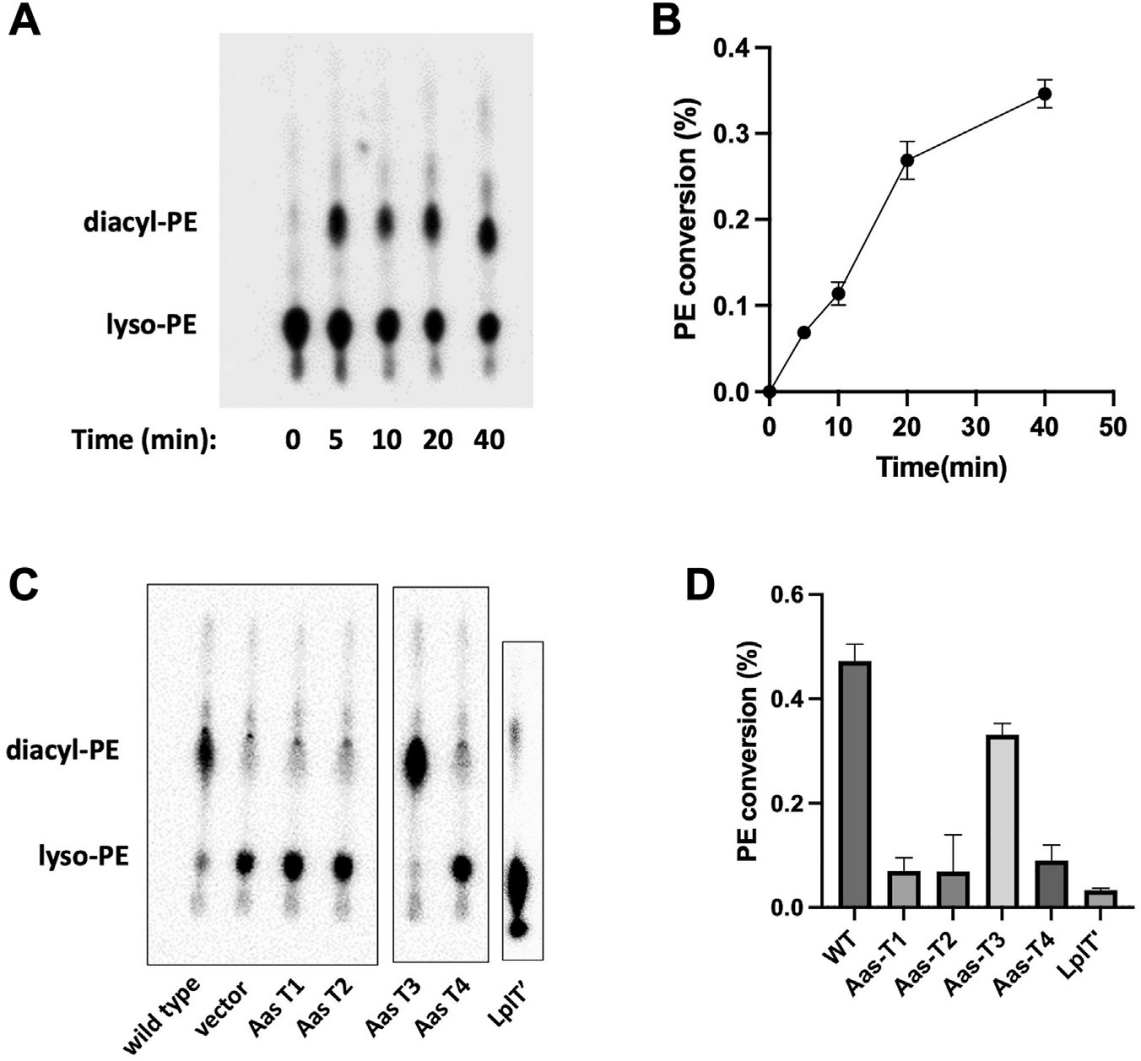
**Essential role of LplT-Aas interaction in lysophospholipid acylation.***A*, a time course of the lyso-PE acylation assays in *Escherichia coli* spheroplasts expressing the operon of Aas-LplT WT or spheroplasts prepared from *E. coli* harboring an empty vector used as a negative control. Subsequently, 10 μM [^32^P] lyso-PE was added into spheroplasts at 37 ^°^C. The reactions were terminated at indicated times by adding chloroform/methanol (1:2). Extracted lipids were separated on Silica gel G thin layer plates and developed with chloroform/methanol/acetic acid (65:25:8, *v/v*). The dried plate was exposed to a storage phosphor screen. Lyso-PE and diacyl-PE were visualized and quantified using Typhoon FLA 9500 and ImageQuant software, respectively. *C*, Lyso-PE acylation assays in *E. coli* spheroplasts expressing LplT-Aas WT, Aas mutants T1-T4, and LplT loop mutant (LplT′). The reaction time is 30 min. Images from different TLC plates are framed using *black squares*. *B* and *D*, quantification of the substrate (lyso-PE) and acylated products (diacyl-PE) are shown in *A* and *C*. Error bars represent standard deviations (n = 3). PE, phosphatidylethanolamine.

Our previous study has shown a strong coupling of LplT and Aas in maintaining the lysophospholipid acylation in *E. coli* (16). To verify the substrate selectivity of LplT in coupled translocation and regeneration reactions, we have previously developed a novel *in vivo* assay using intact spheroplasts (24). In this remodeling assay, lysophospholipid translocation and repair can be demonstrated by quantitively measuring the Aas-dependent acylation of transported radiolabeled lysophospholipids using TLC. By this assay, mutating either LplT or Aas induced similar membrane disruptive effects. We hypothesized that this coupling is synchronized by strong protein-protein interaction. Therefore, we decided to examine the effects of mutations introduced strategically on the predicted protein interface using the acylation assay with radioactive lyso-PE. The assays were performed using spheroplasts generated from *E. coli* cells in which the operon of Aas-LplT of *E. coli* was expressed under the control of a T7 promoter. As seen on the TLC image (Fig. 6, *A* and *B*), [P^32^] lyso-PE was imported into the spheroplasts, which was subsequently acylated to generate diacyl PE in a time-dependent manner. In contrast, no detectable activity was observed in the spheroplasts prepared from *E. coli* cells harboring an empty vector (Fig. 6, *C* and *D*). The interface mutations were then introduced in the operon construct. Consistently with the BACTH results, substituting the central loop of LplT with the counterpart of LacY (LplT′) dramatically reduced the acylation activity compared to the WT as evidenced by the low yield of lyso-PE into PE conversion. Similar disruptive effects were also observed with the Aas mutants lacking T1, T2, or T4 segments. In sharp contrast, no significant change was observed in the mutation of T3. These results reveal that maintaining the direct protein interaction of LplT and Aas is essential for lysophospholipid remodeling in the bacterial envelope.

## Discussion

LplT and Aas proteins provide a unique bacterial membrane phospholipid repair mechanism which is required for maintaining the integrity of the membrane envelope in *E. coli* and possibly other gram-negative bacteria. Transiently residing lysophospholipids in diderm bacteria are translocated back to the inner membrane where they can be taken up by LplT, a translocase component of retrograde transport system that recycles potentially harmful detergent like lysophospholipids back to the cytoplasmic surface of the inner membrane where they can be reacylated by Aas (15, 16). Proper functioning of this tandem system is important to maintaining the stability and integrity of the bacterial envelope (19). In this study, using complimentary *in vivo* BACTH and *in vitro* binding approach we demonstrated that LplT and Aas form *in vivo* a complex with a 1:1 ratio. The strong interaction between LplT and Aas is revealed further by the BLI analysis. These results provide new insight into the working mechanism of this coupled lysolipid scavenging system.

As the only lysolipid scavenging system identified in *E. coli*, LplT-Aas helps to maintain lysophospholipids at very low levels since the membrane bilayer structure is highly susceptible to the detergent effect of lysophospholipids (15). To ensure this effective process, LplT recruits lysophospholipid from the periplasmic space with a high affinity (16). Although LplT has broad substrate specificity, it is well suited to rescue PE, PG, and cardiolipin from their lysoform with comparable kinetics and binding (16). Since flipped lysophospholipids are prone to diffuse passively along the membrane, it is important to understand mechanistically how they are efficiently delivered to Aas for acylation reaction to minimize or even completely exclude the contacts and/or transitions from bulk lipid solvent into the buried catalytic site. Our results indicate that the transferase domain of Aas directly interacts with LplT, and the loops adjacent to the active site play a critical role in mediating protein interaction (Fig. 5). These results suggest that the active site of Aas can be directly attached to LplT. This docking pose may provide a unique mechanism that allows the direct transfer of flipped lysolipids to the catalytic site of Aas for acyl transfer reaction.

The mechanism by which LplT contributes to this complex formation remains elusive. Our results show that both the N-lobe and central cytoplasmic loop are important for the binding to Aas (Fig. 4*C*). The Alphafold model of Aas suggests that hydrophobic interactions play a major role in LplT-Aas interaction since the molecular surface of the transferase domain is highly hydrophobic based on the surface electrostatics map (Fig. 5*B*). However, the central cytoplasmic loop of LplT contains several hydrophilic residues (Fig. 4*B*). It is unlikely that this loop is directly associated with the hydrophobic Aas-T domain. Instead, the interaction may be mediated by the N-lobe domain due to its hydrophobic membrane interface.

LplT is a membrane of the MFS family. MFS proteins generally use a common rock-switching mechanism to transport substrates from one side to another side of the membrane through the central translocation pathway (25). Unlike other MFS members, our previous study has suggested that LplT uses a unique “credit card”-sliding mechanism to flip lysophospholipids (16). We found that LplT recruits substrates *via* two routes, either from its extracellular entry or through a membrane-embedded groove between the two lobes (21). After flipping, the substrate may be released from the lobe interface laterally toward the inner leaflet whereas mutations at the cytoplasmic exit of the putative MFS translocation pathway led to no changes in the LplT transport activity (21). The N-lobe is important for substrate binding and transport; for example, the mutation of D30N completely abolishes transport activity (21). Therefore, Aas may interact with the N-lobe of LplT to take flipped substrates from the substrate translocation pathway to its transferase active site. This hypothesis is supported by our pull-down assays. The presence of lysophospholipid substrates, but not their diacyl counterparts, reduced the interaction of LplT-Aas, implying that substrate binding is involved in the protein interaction (Fig. 3*B*). It is unclear how the mutation within the central cytoplasmic loop disrupted its binding to Aas since this loop is generally less important for the transport activity of MFS proteins (26). However, this loop may be important for assembling the translocation pathway of LplT since it is the only physical link between the two lobes (Fig. 4*A*). The mutation within the loop may alter the conformation of the N-lobe and its interaction with Aas. Our future structural analysis of the LplT-Aas complex will help us reveal any allosteric conformational changes in the protein-protein interaction.

Our results are consistent with a model of the coupling mechanism, in which the translocation pathway of LplT is directly connected with the active site of the transferase domain of Aas. This mechanism integrates LplT and Aas into a unified lysophospholipid scavenging system for efficient repairing of potential lysolipid-induced damage within the bacterial envelope. This stable protein complex may also ensure the lysophospholipid specificity of Aas. A recent study showed that overexpression of Aas alone led to the accumulation of N-acyl PE and N-acyl PG to ∼20% of the total lipid composition in *E. coli* (27). LplT is highly specific for lysophospholipids (16). The coupling mechanism may prevent Aas access to bulk diacyl phospholipids to prevent an “accidental” formation of potentially harmful membranes, therefore maintaining the integrity of the bacterial membranes.

Although LplT-Aas is only identified in gram-negative bacteria (15), we cannot exclude that other lysophospholipid transporters are also available in other microorganisms. A similar lysophospholipid uptake/acylation activity has been reported in gram-positive bacteria; for example, several species of *Streptococcus* including *Streptococcus pneumoniae* scavenge lyso-phosphatidylcholine (lyso-PC) from human sera, which was acylated to form PC (28). MFS is the largest membrane protein family and several hundred homologs have been identified in many cell types (29). These homologs adapt a great substrate diversity, and their substrates include ions, metabolites, nucleotides, lipids, and even large peptides. It is possible that some MFS homologs also promote lysophospholipid transport. To support this possibility, MFSD2a, a mammalian MFS homolog promotes Na^+^-dependent uptake of lyso-PC and plays an important role in docosahexaenoic acid supplement to the brain and maintaining the stability of the brain–blood barrier (30, 31). Although MFSD2a shares no sequence homology with bacterial LplT, recent studies suggest that these two proteins share a similar flipping mechanism (21, 32). Furthermore, a possible acyl transfer reaction was predicted in the downstream processes of lyso-PC uptake (33). Therefore, the mechanism of the LplT-Aas axis may be presented broadly in different domains of life. The biochemical characterization of the LplT-Aas protein complex reported here opens the door to analyses of coupled lipid flipping and regeneration mechanisms in different organisms.

## Experimental procedures

### Chemicals

H_3_[^32^P]PO_4_ was obtained from MP Biomedicals. 1-Oleoyl-2-hydroxy-sn-glycero-3-phosphoethanolamine (18:1 lyso-PE), POPE, POPG, cardiolipin, and *E. coli* polar lipid extract were purchased from Avanti Polar Lipids. Purified PLA_2_ from *Crotalus adamanteus* venom was from Worthington. Restriction endonucleases, T4 DNA ligase, and Phusion DNA polymerase were from Thermo Fisher Scientific or New England Biolabs. Oligodeoxynucleotides were custom-synthesized by Sigma-Genosys. DDM was from Anatrace.

### Plasmid and strain construction

DNA encoding for Aas, LplT, and the tandem Aas-LplT was amplified by PCR from genomic DNA of *E. coli* W3110 using the appropriate oligonucleotides and cloned into pET28a(+); generating the overexpression plasmids pET28a-Aas, pET28a-LplT, and pET28a-Aas/LplT. Aas or LplT was expressed as N-terminal 6His tagged fusion proteins, respectively. No fusion tag was placed in the operon construct. For two-hybrid assays, the gene of *lplT* or *aas* was cloned to pUT18C or pKT25 to express T18 or T25 fusion proteins, respectively. Mutations were generated using a modified QuikChange site-directed mutagenesis approach (34). All constructs were confirmed by sequencing.

### Bacterial two-hybrid assay

Protein interactions were tested using the BACTH (Euromedex, France). For each assay, plasmid pairs encoding T18 and T25 fusion proteins were introduced into *E. coli* BTH101 and plated onto LB agar supplemented with 100 μg ml^−1^ ampicillin and 50 μg/ml kanamycin, 40 μg/ml X-Gal, and 1 mM IPTG. Three to five transformants were inoculated into 1 ml of LB containing 100 μg/ml ampicillin and 50 μg/ml kanamycin and incubated for at least 4 h at 37 °C. Five microliters of each culture was then spotted onto prewarmed X-Gal agar containing both antibiotics. Positive interactions were identified as blue colonies on X-Gal LB agar after incubating plates for 48 h at 30 °C. For each plasmid pair, triplicate experiments were performed.

### β-galactosidase assays

The strength of protein interaction was quantified by β-galactosidase assays using a published protocol (35). Cotransformants were grown overnight in LB broth with 100 μg/ml ampicillin and 50 μg/ml kanamycin, 50 μl of each culture was transferred to a 96-well flat-bottom microtiter plate containing 150 μl of sterile water in each well. The *A*_600_ of the diluted culture was determined by plate reader (BioTek). For activity assay, 50 μl of each culture was mixed with 500 μl Z buffer (60 mM Na_2_HPO_4_, 40 mM NaH_2_PO_4_, 10 mM KCl, 1 mM MgSO_4_, and 50 mM β-mercaptoethanol) before adding 20 μl of freshly prepared 0.1% SDS and 40 μl of chloroform. For permeabilization, aspirating and dispensing the mixtures 10 to 15 times with a pipette. After 10 min, transfer 100 μl upper solution of permeabilized cells in Z-buffer to another 96-well flat-bottom microtiter plate, the assay is initiated by adding 20 μl of 4 mg/ml ortho-nitrophenyl-β-galactoside to each well of the microplate. After 10 min, 50 μl of 1 M Na_2_CO_3_ was added to terminate the reaction, and then A_420_ was measured by plate reader. The relative β-galactosidase activity of each sample was expressed as nmoles of β-galactose formed per minute per mg of the lysate (a value of 1.4 *A*_600_ is approximately 0.150 mg protein, and 1 nmol/ml ortho-nitrophenyl-β-galactoside corresponds to 0.0045 *A*_420_). All assays were performed in triplicate, and results were expressed as the mean ± standard deviation.

### Protein expression and purification

The LplT and Aas proteins were expressed and purified using similar approaches (16). Briefly, protein expression was carried out in *E. coli* BL21(DE3) strain in autoinduction medium (36) for 3 h at 37 °C followed by overnight incubation at 25 °C. Cells were harvested by centrifugation and resuspended in lysis buffer containing 500 mM NaCl, 50 mm Tris–HCl, pH 8.0, 10 mM imidazole, and 10% glycerol. The cell membrane was disrupted by passing through an Avestin H3 homogenizer at 15,000 p.s.i. Cell debris was removed by centrifugation at 16,000 rpm for 30 min using an S34 rotor (Beckman Coulter). The supernatant was collected and ultracentrifuged at 40,000 rpm for 1 h using a Ti45 rotor (Beckman Coulter). The pellet containing the membrane fraction was resuspended in lysis buffer and then incubated with 1% (w/v) DDM for 1 h at 4 °C. After another ultracentrifugation step at 40,000 rpm for 30 min, the supernatant was incubated with Ni-NTA affinity resin at 4 °C for 1 h in an orbital shaker. The Ni^2+^-NTA affinity resin was washed with lysis buffer containing 0.03% DDM and 40 mM imidazole. The proteins were eluted in lysis buffer with 400 mM imidazole and further desalted by a desalting column in a buffer containing 100 mM NaCl, 20 mM Tris–HCl, pH 7.5, and 0.03% DDM. To generate tag-free LplT, thrombin digestion cleavages the N-terminal His tag followed by filtration through a Ni-NTA column. The filtrate containing tag-free LplT was collected and used for binding assays.

### Pull-down assay

Proteins were mixed in 200 μl of binding buffer (10 mM Hepes/NaOH, 300 mM NaCl, 0.03% DDM, pH 7.5). The protein concentrations were 1.5 or 2.0 μM for LplT or Aas. Samples were incubated at ambient temperature for 10 min to allow possible complexes to form. Briefly, 2 mM lyso-PE, POPE, POPG, cardiolipin, or *E. coli* total lipid extract solubilized in 1% DDM was added to the solution, respectively. Complexes were pulled down by O/N incubation at 4 °C with 100 μl of washed and equilibrated Ni-NTA superflow beads (QIAGEN). Beads were washed 5 to 8 times with 1.5 ml of wash buffer (10 mM Hepes/NaOH pH 7.5, 10 mM MgCl_2_, 500 mM NaCl, 50 mM imidazole, and 0.03% DDM). Retained proteins were eluted by 250 mM imidazole and then were resolved by SDS-PAGE.

### Bilayer interferometry

Binding kinetics was characterized on a ForteBio Octet RED96 system (Pall). Before assays, all proteins were desalted into the buffer: 25 mM Tris–HCl (pH 7.4), 200 mM NaCl, 0.05% DDM, using a desalting column. Aas protein (0.375 μM) was loaded onto the NTA-Ni biosensors for 300 s. Following 180s of baseline in the same assay buffer, the loaded biosensors were dipped into serially diluted LplT protein for 300 s. The sensors were then dipped into the buffer for 600 s to record dissociation kinetics. Buffer without protein was set to correct the background. Data collection was performed using the Octet Data Acquisition 9.0 software (Pall). For fitting of *K*_D_ value, Octet Data Analysis software V11.1 (Pall) was used to fit the curve by a 1:1 binding model and use the global fitting method.

### Preparation of radiolabeled lyso-PE

Radiolabeled lyso-PE was prepared by digestion of purified [^32^P] PE with venom PLA_2_ essentially as we described (16). [^32^P] PE was synthesized in *E. coli* strain UE54 (MG1655 lpp-2Δara714 rcsF::mini-Tn10 cam pgsA::FRT-kan-FRT) (37). The strain accumulates a level of PE to 95% in deep stationary phase cells. [^32^P] PE was produced by growing UE54 cells in 50 ml of LB medium containing 5 μCi/ml [^32^P] PO_4_ overnight. The cells were harvested for lipid extraction using the Bligh and Dyer method as described (24). To generate [^32^P] lyso-PE, dried [^32^P] PE was dissolved in 0.5 ml digestion solution (0.1 M Hepes-NaOH pH 7.5, 0.1 M KCl, 10 mM CaCl_2,_ and 1% DDM). The reactions were incubated at 37 °C overnight with shaking. After incubation, lipids were extracted and loaded onto Silica Gel G thin-layer plates and developed with chloroform: methanol: acetic acid (65:25:8 v/v). The bands corresponding to lyso-PE on the TLC plate were scraped and extracted using chloroform. Purified [^32^P] lyso-PE was kept at −20 °C before assays.

### Lysophospholipid acylation assays

Lysophospholipid acylation assays were performed in freshly prepared spheroplasts prepared according to published protocols with slight modifications (16). *E. coli* BL21(DE3) expressing Aas/LplT WT or mutants were grown in LB broth at 37 °C, 0.2 mM IPTG was added when *A*_600_ reached 0.6, then induced at 25 °C for 2 h. Cells were pelleted, washed twice, and resuspended in 10 mM Hepes, pH 7.5, 0.75 M sucrose, 10 mM MgSO_4_, 2.5% (*w/v*) LiCl. After the addition of 1 mg/ml lysozyme, cell suspensions were ice-chilled for 5 min. Intact spheroplasts were collected by centrifugation (3000*g* for 10 min) at room temperature and resuspended at 10 mg/ml total protein in the above buffer without LiCl. Spheroplast formation and stability were thoroughly monitored nephelometrically by comparing the *A*_600_ of a 100 μl spheroplast solution with 2 ml of either plain water or a solution of 10 mM MgCl_2_, 0.75 M sucrose, respectively.

Prior to the transport assay, 1000 cpm of [^32^P] lyso-PE was mixed with synthetic 18:1 lyso-PE and resuspended in ethanol to a final concentration of 200 μM. The reactions were initiated by adding 10 μM (final concentration) substrates into spheroplast solutions. At the indicated time, reactions were terminated by adding chloroform/methanol (1:2), and total lipids were extracted and separated on Silica Gel G TLC plates (Agela Technologies) with a solvent consisting of chloroform: methanol: acetic acid (65:25:8 *v/v*). The dry plate was exposed to a storage phosphor screen for 2 days. Radiolabeled lipids were visualized using a Typhoon FLA 9500 Imager (Cytiva Inc). Stored images were processed and quantified using ImageQuant software (Cytiva Inc). Phospholipid content is expressed as mol % of total phospholipid based on the intensity of the captured signal generating a latent image of the radiolabeled spot on the Phosphor Screen.

## Data availability

All data are contained within the manuscript.

## Conflict of interest

The authors (L. Z. and M. B.) are Editorial Board Members for *the Journal of Biological Chemistry* and were not involved in the editorial review or the decision to publish this article. The other authors declare that they have no conflicts of interest with the contents of this article.
